# CNS4 causes subtype‐specific changes in agonist efficacy and reversal potential of permeant cations in NMDA receptors

**DOI:** 10.1002/prp2.1107

**Published:** 2023-06-07

**Authors:** Seth C. Boehringer, Tulia V. Johnston, Lina Cortez Kwapisz, Pamela J. VandeVord, Blaise M. Costa

**Affiliations:** ^1^ Edward Via Virginia College of Osteopathic Medicine Virginia Blacksburg USA; ^2^ Department of Biochemistry Virginia Tech Blacksburg Virginia USA; ^3^ Department of Biomedical Sciences and Pathobiology Virginia‐Maryland College of Veterinary Medicine Virginia Blacksburg USA; ^4^ Department of Biomedical Engineering and Mechanics Virginia Tech Blacksburg Virginia USA; ^5^ Center for One Health Research, Virginia Tech Virginia Blacksburg USA

**Keywords:** glutamate, GRIN disorder, hyperglutamate, hypoglutamate, neuropsychiatric disorder, NMDA receptor

## Abstract

The NMDA subtype of glutamate receptor serves as an attractive drug target for the treatment of disorders evolving from hyper‐ or hypoglutamatergic conditions. Compounds that optimize the function of NMDA receptors are of great clinical significance. Here, we present the pharmacological characterization of a biased allosteric modulator, CNS4. Results indicate that CNS4 sensitizes ambient levels of agonists and reduces higher‐concentration glycine & glutamate efficacy in 1/2AB receptors, but minimally alters these parameters in diheteromeric 1/2A or 1/2B receptors. Glycine efficacy is increased in both 1/2C and 1/2D, while glutamate efficacy is decreased in 1/2C and unaltered in 1/2D. CNS4 does not affect the activity of competitive antagonist binding at glycine (DCKA) and glutamate (DL‐AP5) sites; however, it decreases memantine potency in 1/2A receptors but not in 1/2D receptors. Current–voltage (I‐V) relationship studies indicate that CNS4 potentiates 1/2A inward currents, a phenomenon that was reversed in the absence of permeable Na^+^ ions. In 1/2D receptors, CNS4 blocks inward currents based on extracellular Ca^2+^ concentration. Further, CNS4 positively modulates glutamate potency on E781A_1/2A mutant receptors, indicating its role at the distal end of the 1/2A agonist binding domain interface. Together, these findings reveal that CNS4 sensitizes ambient agonists and allosterically modulates agonist efficacy by altering Na^+^ permeability based on the GluN2 subunit composition. Overall, the pharmacology of CNS4 aligns with the need for drug candidates to treat hypoglutamatergic neuropsychiatric conditions such as loss function GRIN disorders and anti‐NMDA receptor encephalitis.

## Graphical abstract

Working hypothesis of CNS4 mechanism of action: Changes in glutamate concentration alter agonist binding domain (ABD) intersubunit interaction, that plays a crucial role in desensitization leading to channel constriction. CNS4 (blue) preferentially reverses the low glutamate (broken heart) induced changes in ABD conformation back to normal glutamate‐like ABD conformation facilitating cation influx through the NMDA receptor channel. This will be experimentally evaluated in the future.

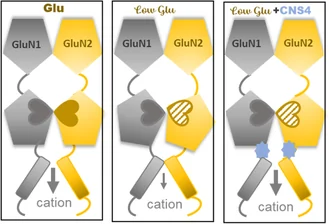

**Abbreviations**
Ca^2+^
: calcium ions
cDNA: complementary DNA
E18: embryonic day‐18
GluN: glutamate receptor NMDA subtype
HEK293T: human embryonic kidney 293 transfectable
Na^+^
: sodium ions
NMDA: N‐methyl D‐aspartate
TEVC: two electrode voltage clamp

## 1 INTRODUCTION

Approximately 8000 glutamate molecules are released from a synaptic vesicle with an average diameter of ~40 nm.^1^, ^2^ These glutamate molecules are reconstituted into 20–30 nL volume of extracellular fluid in the synaptic cleft, bringing the glutamate concentration to ~1 mM for less than a millisecond and dropping as low as 20 nM before the subsequent glutamate release.^3^, ^4^, ^5^, ^6^, ^7^, ^8^ Glutamate binds with a family of metabotropic and ionotropic glutamate receptors to initiate and propagate excitatory neurotransmission.

The N‐methyl‐D‐Aspartate (NMDA) receptor is a subtype of ionotropic glutamate receptor. There are seven different GluN genes (GluN1, GluN2A, GluN2B, GluN2C, GluN2D, and GluN3A, GluN3B) encoding NMDA receptor subunits. Functional NMDA receptors are hetero‐tetramers composed of two obligatory glycine‐binding GluN1 subunits and one or two GluN2 or GluN3 subunits. Different spatiotemporal expression of GluN2 & 3 subunits enables the heterogeneity needed to elicit subtype‐specific physiological properties.^9^, ^10^, ^11^ Although 1/2A and 1/2B diheteromeric receptors are the canonical representatives, recent studies demonstrate that 1/2AB containing tri‐heteromeric NMDA receptors are predominantly expressed throughout the hippocampus and cortex.^12^, ^13^, ^14^, ^15^, ^16^, ^17^

NMDA receptor subtypes with lower glutamate potency (1/2A, EC_50_ ~ 3 μM) are expressed in the synapse, as opposed to the subtypes that exhibit six times higher glutamate potency (1/2D, EC_50_ ~ 0.5 μM) localized at the extrasynaptic sites.^18^, ^19^, ^20^ 1/2C and 1/2D are also expressed in the GABAergic interneurons.^21^ NMDA receptors (GluN1/2) exhibit deactivation time constants that span about a 50‐fold range, with the following order (from fastest to slowest): 2A < 2C < 2B < 2D.^22^ The 1/2A subunit‐containing NMDA receptor deactivation time constant is about ~50 ms; 1/2B, ~400 ms; 1/2C, ~290 ms; and 1/2D, >1 s.^22^ Thus, glutamate spillover through the extrasynaptic 1/2D receptors produces a long‐lasting cation influx into the postsynaptic neurons.

NMDA receptor dysfunction is a major cause for the pathogenesis of psychiatric disorders, in addition to the well‐studied dopamine hypothesis.^23^, ^24^ There are genetic, biochemical and pharmacologic lines of evidence supporting the contribution of NMDA receptor hypofunction to the pathophysiology of schizophrenia^25^ and the comorbidity of substance abuse.^26^ Further, it was recently estimated that one in 5026 people in the United States could have a rare developmental neuropsychiatric condition recently recognized as GRIN (Glutamate Receptor Ionotropic NMDA) disorder.^27^ Majority of the GRIN patients are less than 12‐year‐old. The clinical presentation of GRIN disorders include ADHD, developmental delay, intellectual disability, epilepsy, autism spectrum disorders and movement disorders.^28^ Currently, there is no cure for this condition. GRIN disorder‐causing amino acid mutations in the polypeptide chains of NMDA receptor subunits are reported in the Center for Functional Evaluation of Rare Variants (CFERV) database. According to details provided at CFERV, most patients have loss‐of‐function mutations leading to hypoglutamatergic neurotransmission. Therefore, drugs that selectively modulate hypoactive NMDA receptors could be used to treat GRIN disorders with reduced glutamate potency. In this study, we performed pharmacological characterization of a small molecule (CNS4) that modulates NMDA receptor function based on GluN2 subunit composition and agonist concentration.

## 2 MATERIALS AND METHODS

### 2.1 HEK‐293T cells & whole‐cell patch‐clamp electrophysiology

**FIGURE 1:**
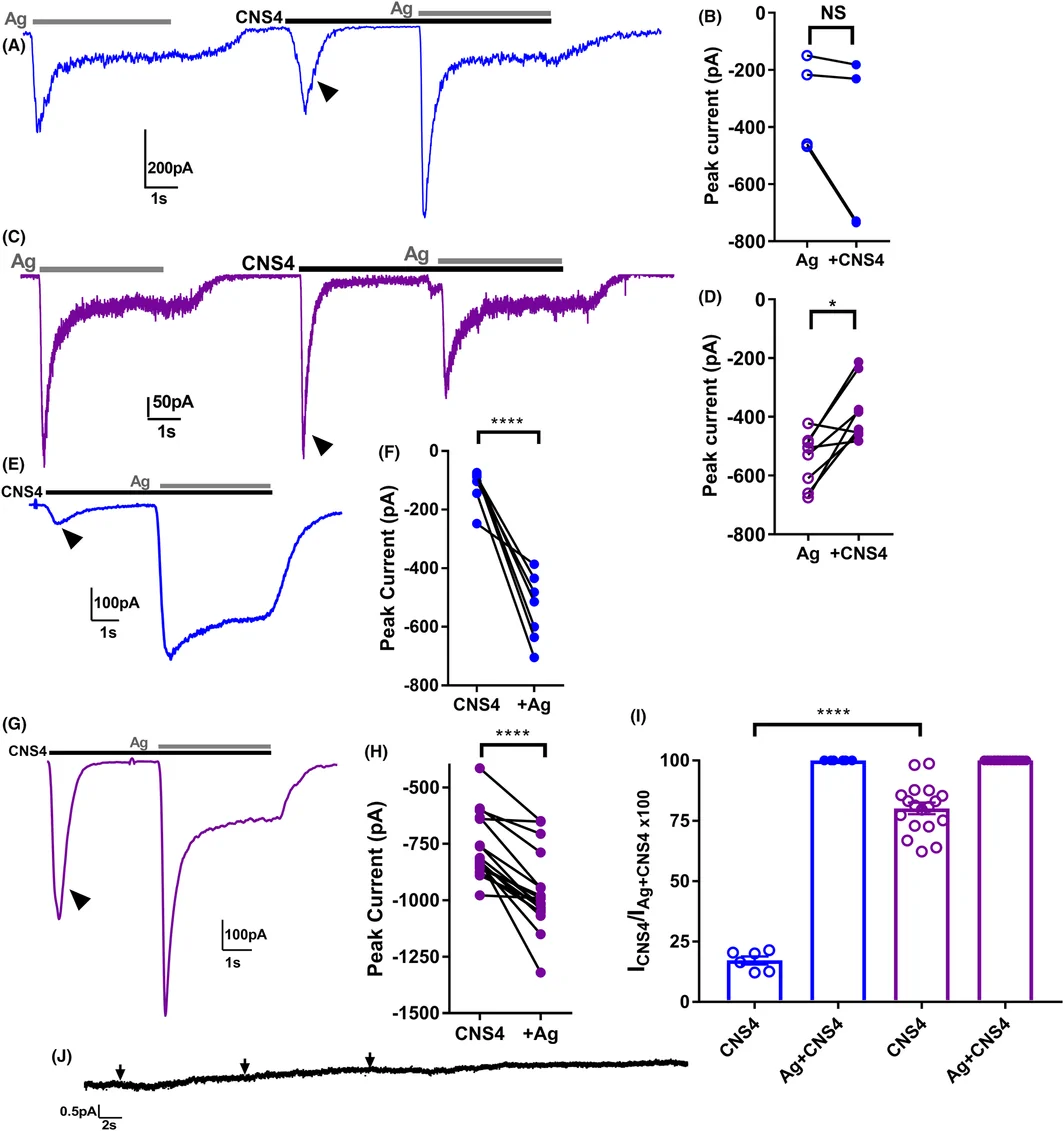
CNS4 sensitizes ambient concentration of agonists in GluN2 subunit dependent manner. Whole cell patch‐clamp electrophysiology assays performed using HEK293T cells co‐expressing recombinant GluN1/2A (blue, A&E) and GluN1/2AB (purple, C&G) receptors. Traces represent current responses evoked by 100 μM glutamate and 100 μM glycine as agonists (Ag, gray bar). Black bar indicates application of 100 μM CNS4 4 s before and co‐application with agonists (+CNS4). Peak current amplitude values were used for the analysis. CNS4 pre‐application evoked a transient and completely reversible current (marked with arrowhead) both with GluN1/2A and 1/2AB receptors. Each pair of Ag and +CNS4 application events is shown in connected dot plots (B&D). In 1/2A, Ag, −323.5 ± 82 pA versus +CNS4, −469.3 ± 152 pA, *n* = 4(3), *p* = .12. 1/2AB, Ag, −546 ± 32.34 pA versus +CNS4–380.7 ± 36.61 pA, *n* = 8(4), *p* = .023. E&G, There was no agonist alone application in this set experiments. CNS4 (in the recording buffer) was applied for 4 s before co‐application with Ag. Each pair of CNS4 and CNS4 + Ag (+Ag) application events are shown in connected dot plots (F&H). I, CNS4 + Ag induced current response was normalized to one hundred to calculate relative percentage current amplitudes. CNS4 alone application induced transient current was 17.24 ± 1.6%, *n* = 7 (3), of CNS4 + Ag current (100%) in GluN1/2A receptors. However, in GluN1/2AB receptors (G&H), this was as high as 80.15 ± 2.3%, *n* = 18 (4), of maximal current amplitude. Statistical significance was calculated from the Wilcoxon matched‐pairs signed rank test, except the comparison between 1/2A & 1/2AB CNS4 with no agonist data, which was done by unpaired *t*‐test. (I). *p < .05, **p < .01, ****p < .0001. 100 μM Glu, 1/2A, *n* = 4, 1/2AB, *n* = 8 pairs. NS, not significant. (J) Trace shows similar whole cell patch clamp assay on an untransfected HEK293T cell, where CNS4 produced no current response. Downward arrows represent CNS4 application points.

Whole‐cell patch‐clamp electrophysiology studies were carried out in HEK‐293T cells expressing recombinant NMDA receptors that lack native functional NMDA receptors.^29^, ^30^, ^31^ An equal quantity of (1 μg) cDNA for GluN1a, GluN2 subunits were co‐transfected 24‐48 h before patch‐clamp electrophysiology assay. Activation of NMDA receptors by ambient glutamate from the cell culture media was inhibited (to avoid excitotoxicity) by adding 50 μM memantine into the culture media during transfection.^32^ Cells were carefully washed before performing experiments and were used for the electrophysiology experiments after 24 to 48 h incubation at 37°C with 5%CO_2_. The whole‐cell patch‐clamp electrophysiology assay was performed using semi‐automated patch‐clamp equipment, Port‐a‐Patch (Nanion Technologies GmbH). Following are the constituents of various solutions used for patch‐clamp electrophysiology: internal solution ([mM] NaCl 10, EGTA 20, CsF 110, HEPES 10), Mg‐free external (recording) solution ([mM] NaCl 140, KCl 4, CaCl_2_ 2, HEPES 10, D‐GlucoseMonohydrate 5), and Mg‐free seal enhancer solution ([mM] NaCl 80, KCl 3, CaCl_2_ 35, HEPES 10). Nanion NPC chips with 2–3.5 mOhms resistance were used for the HEK‐293T cell recordings. Agonist concentrations used for this set of experiments are provided in the Figure 1 caption.

### 2.2 Two electrode voltage clamp (TEVC) electrophysiology using Xenopus oocytes

NMDA receptor constructs: cDNA encoding the NMDA receptor1a subunit (GluN1a) was obtained from Dr. Nakanishi (Kyoto, Japan). cDNA encoding the GluN2B (pci_sepGluN2B), originally developed in the Malinow lab,^33^ was purchased from Addgene (cat# 23998), Cambridge, MA. cDNA encoding the GluN2C and GluN2D were purchased from GenScript, New Jersey, USA. Mutated GluN1, 2A and 2B cDNA constructs capable of assembling as GluN1/2A/2B triheteromeric (1/2AB) receptors^34^ were obtained from Dr. Paoletti (Laboratoire de Neurobiologie, CNRS). These constructs have been previously tested for 1/2AB receptor activity of GluN1/2A receptor‐selective potentiators.^35^ GluN1 mutants (N521A, N521D, K531A, Y535A, and E781A) were generated by site‐directed mutagenesis (QuikChange XL site‐directed mutagenesis kit; Stratagene) and confirmed by DNA sequencing. Plasmids were linearized with NotI (GluN1a and all five GluN1 mutants) or Avrll (GluN2B), or BstB1 (GluN2C and GluN2D) and transcribed in vitro with T7 (GluN1a, GluN1/2A, GluN2B, GluN2C & GluN2D), and SP6 (GluN1 mutants) RNA polymerase using the mMessage mMachine transcription kits (Invitrogen by Thermo Fisher Scientific).

### 2.3 GluN subunit expression and electrophysiology in Xenopus oocytes

Stage IV frog oocytes were obtained from Xenopus‐I, (Ann Arbor, MI, USA). NMDA receptor subunit cRNAs were suspended in nuclease free sterile water. GluN1A, GluN2B, GluN2C, GluN2D and GluN1/2A/2B cRNAs were mixed in a ratio of 1:1–3. 50 nL of the final cRNA mixture was microinjected (40–70 ng total) into the oocyte cytoplasm. Oocytes were incubated in ND‐96 solution at 18°C prior to electrophysiological recordings (2–5 days).

### 2.4 Dose–response curves

**FIGURE 2:**
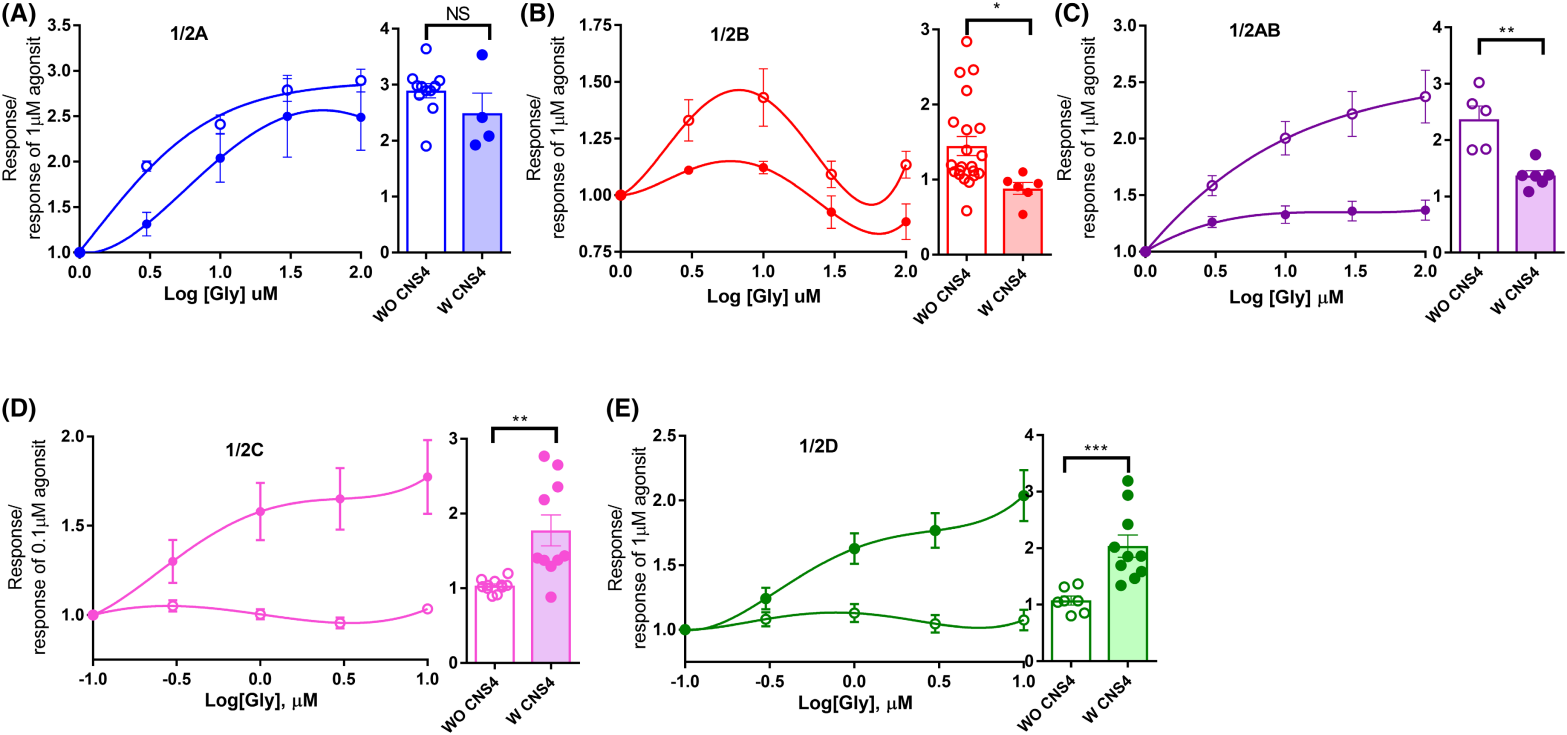
CNS4 enhances the glycine efficacy in 1/2C & 1/2D but reduces in 1/2B, and 1/2AB receptors. Glycine dose–response curves for 1/2A, 1/2B, 1/2AB, 1/2C and 1/2D subtypes of NMDA receptors in the absence (empty circles) and presence (filled circles) of 30 μM CNS4 were performed. 100 μM glutamate was kept constant. A relative increase in the current response after 1 μM (or 100 nM for 2C & 2D) glycine was calculated and plotted. Histograms summarize relative fold change in the highest concentration of glycine studied. Glycine efficacy changes without (WO) versus with (W) CNS4 is compared: 1/2A (2.89 ± 0.11, *n* = 11 (4) vs. 2.59 ± 0.36, *n* = 4 (4), *p* = .19); 1/2B (1.45 ± 0.13, *n* = 21 (13) vs. 0.88 ± 0.07, *n* = 6 (6), *p* = .0303)); and 1/2AB (2.37 ± 0.23, *n* = 5 (4) vs. 1.37 ± 0.08, *n* = 6 (4), *p* = .0019); 1/2C (1.03 ± 0.02, *n* = 11 (5) vs. 1.77 ± 0.20, *n* = 10 (5), *p* = .0015); and 1/2D (1.075 ± 0.07, *n* = 7 (3) vs. 2.04 ± 0.19, *n* = 10 (5) *p* = .0014). Values are average ± SEM. Unpaired student's *t*‐test, *p* < .05. *p < .05, **p < .01 and ***p < .001. NS, not significant.

**FIGURE 3:**
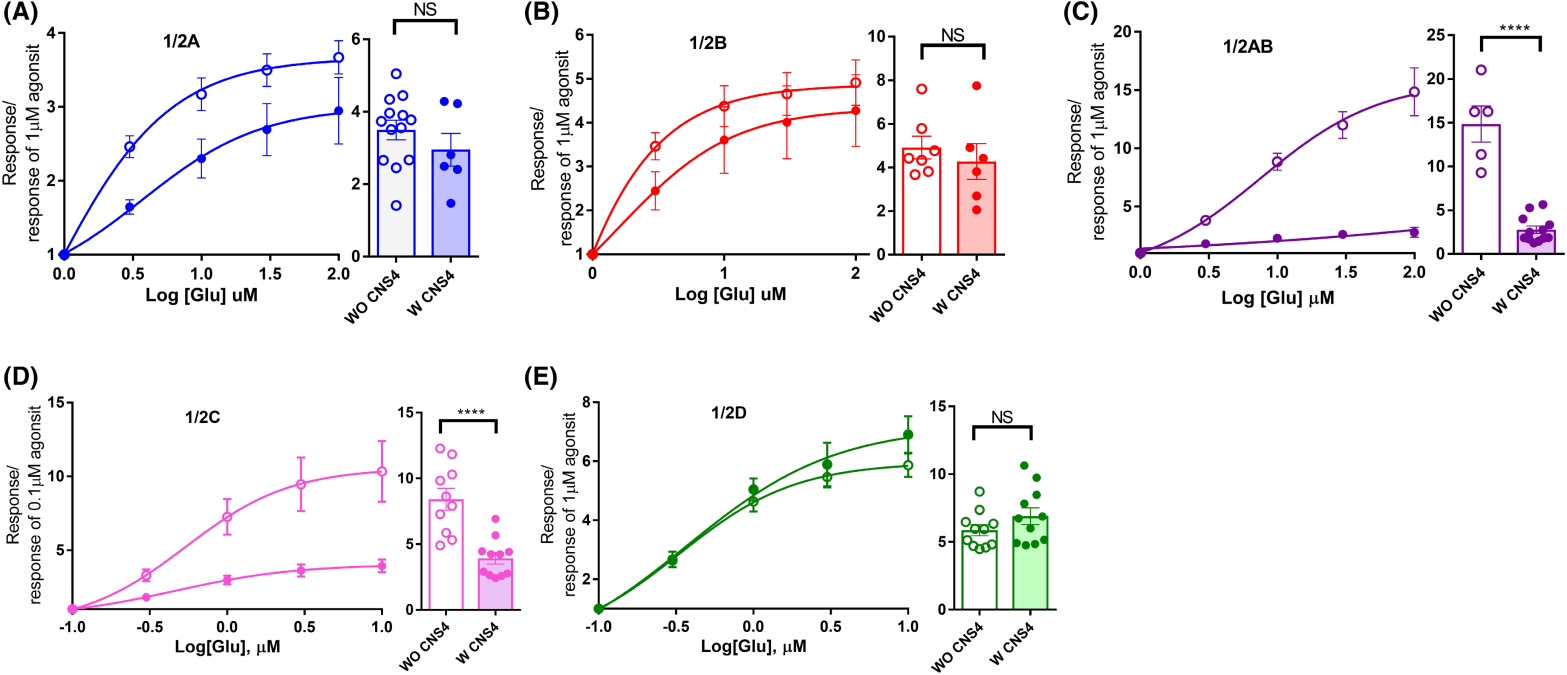
CNS4 reduces glutamate efficacy in 1/2AB, 1/2C but unaltered 1/2A, 1/2B, and 1/2D receptors. Glutamate dose–response curves for 1/2A, 1/2B, 1/2AB, 1/2C and 1/2D subtypes of NMDA receptors in the absence (empty circles) and presence (filled circles) of 30 μM CNS4 were performed. 100 μM glycine was kept constant. A relative increase in the current response after 1 μM (or 100 nM for 2C & 2D) glutamate was calculated and plotted. Histograms summarize relative fold change in the highest concentration of glutamate studied. Glutamate efficacy changes without (WO) versus with (W) CNS4 is compared: 1/2A (3.45 ± 0.27, *n* = 13 (5) vs. 2.95 ± 0.45, *n* = 6 (6), *p* = .2898), 1/2B (4.91 ± 0.52, *n* = 7 (4) vs. 4.27 ± 0.82, *n* = 6 (4), *p* = .5104), and 1/2AB (14.85 ± 2.06, *n* = 5 (4) vs. 2.79 ± 0.43, *n* = 12 (6), *p* = .0001), 1/2C (8.41 ± 0.83, *n* = 10 (5) vs. 3.93 ± 0.43, *n* = 11(5), *p* = .0001, and 1/2D (5.87 ± 0.40, *n* = 11 (5) vs. 6.90 ± 0.62, *n* = 11 (5), *p* = .1735). Values are average ± SEM. Unpaired student's *t*‐test, *p* < .05. *p < .05, **p < .01 and ****p < .0001.

Electrophysiological responses were measured using a standard two‐microelectrode voltage‐clamp workstation (Warner Instruments model OC‐725C) designed to provide a fast clamp of large cells. The recording buffer contained 116 mM NaCl, 2 mM KCl, 0.3 mM BaCl2, and 5 mM HEPES, pH 7.4. Response magnitude was determined in Xenopus oocytes expressing different NMDA receptor subunits (GluN1/2A, 1/2B, 1/2C, 1/2D, and 1/2A/2B) by the steady plateau response elicited by bath application of different agonist concentrations. And 100 μM L‐glutamate and 100 μM glycine were used to activate the receptors at a holding potential of −60 mV. Response amplitudes for functional NMDA receptors were generally between 0.1 and 2 μA. And 30 μM CNS4 and 100 μM glutamate (or glycine) agonist doses were applied on the oocytes using an 8‐channel perfusion system (Automate Scientific), and the responses were digitized for quantification (Digidata 1550A and pClamp‐10, Molecular Devices). All data points were obtained from the steady state currents unless stated otherwise. Dose–response relationships were fit to an appropriate curve‐fitting equation using GraphPad Prism‐7. Non‐linear regression was used to calculate EC_50_, IC_50_, and fold change or percentage maximal response. Curves were fit using the following equations as performed in the previous studies^36^, ^37^: $\begin{array}{c} Y=100/\left(1+10^{\left(\left({\text{LogEC}}_{50}-X\right)\right)}\right)\;\text{or}\;Y=\text{Bottom}+\left(\mathrm{Top}-\text{Bottom}\right)/\left(1+10^{\left(\left({\text{LogEC}}_{50}-X\right)\right)}\right) \end{array}$. Statistical significance was determined at the overall alpha level 0.05, using appropriate statistical methods as described in all figure captions. Technical and biological replicates are explicitly mentioned in each figure legend, as *n* = X (Y). X represents technical repeats (number of traces) and Y represents biological repeats (number of oocytes or HEK293T cells studied). For CNS4 efficacy plots, currents evoked by 1 μM concentration of glycine or glutamate were normalized to one and we calculated the relative currents evoked by higher concentrations of agonists. Although lower doses (0.1 μM and 0.3 uM) were used in the experiment, 1 μM current was set as the lowest data point because currents evoked by the lower agonist concentrations had higher variability. This variability, when scaled up to currents evoked by higher doses, increased the standard deviation by orders of magnitude and complicated the calculations. Therefore, 1 μM glycine (or glutamate) data sets were normalized to one (in 1/2A, 1/2B & 1/2AB receptors) and the current response from higher concentrations was plotted in Figures 2 and 3. NMDA receptor antagonists were obtained from Tocris Bioscience, Bristol, UK (DL‐AP5, cat# 76326‐31‐3; Memantine, cat # 19982‐08‐2; 5,7‐DCKA cat#13112376‐7). For the data sets with nonparametric distribution, Wilcoxon signed rank test is a nonparametric test was used (Figure 1). Paired or unpaired t‐tests were for used only compare the datasets obtained with and without CNS4 dose response curves. Dose dependent increase in current response was fitted into a sigmoidal curve using appropriate fitting algorithms.

### 2.5 Current–voltage (I‐V) relationship experiments

There are two sets of recording solutions used for the I‐V assay: solutions with normal (1) and low (2) Ca^2+^ concentrations. The normal Ca^2+^ solutions contained (mM): 115 NaCl, 1 KCl, 1.8 CaCl_2_, 10 HEPES. PH was adjusted to 7.2. Low calcium solutions contained 0.018 mM CaCl_2_ and other components were the same as normal Ca^2+^ solution. To study the effects of CNS4 in the absence of extracellular Na^+^, NaCl was replaced with impermeable agent, N‐methyl‐D‐glucamine (NMDG, 230 mM). HCl was used to dissolve the NMDG and to adjust the PH to 7.2. NMDG‐containing solutions are termed Na_free_. The I‐V relationship was studied using Xenopus oocytes expressing 1/2A and 1/2D receptors using 100 μM glutamate + 100 μM glycine agonists (with or without 100 μM CNS4) application at different holding potentials starting from −90 mV up to +40 mV in 10 mV intervals. There were four different agonist solutions used for each set of Ca^2+^ recordings: (1) Agonist, (2) Agonist+CNS4, (3) Agonist in Na_free_ solution, (4) Agonist+CNS4 in Na_free_ solution. Each solution was applied to the oocytes in sequential order and the same order was followed for all I‐V recordings. Low Ca^2+^ experiments were carried out in the same way as normal Ca^2+^ experiments, except that they had 0.018 mM Ca^2+^ in all four agonist and recording solutions. Steady state current responses evoked by these solutions at each voltage step were measured. Reversal potentials (E_rev_) were obtained from x‐intercept values when y = 0 after fitting the I‐V data using a third order polynomial, non‐linear curve fitting equation $\left(Y=\text{B0}+B1^{*}X+B2^{*}X^{2}+B3^{*}X^{3}\right)$. E_rev_ obtained from each oocyte with different agonists were compared in a pairwise manner. These differences were then averaged. Paired or unpaired student *t*‐test was performed to compare the data as mentioned in each figure legend.

### 2.6 Nomenclature of targets and ligands

Key protein targets and ligands in this article are hyperlinked to corresponding entries in http://www.guidetopharmacology.org, the common portal for data from the IUPHAR/BPS Guide to PHARMACOLOGY,^38^ and are permanently archived in the Concise Guide to PHARMACOLOGY 2019/20.^39^

## 3 RESULTS

### 3.1 CNS4 sensitizes ambient concentration of agonists in GluN2 subunit dependent manner

To demonstrate the GluN2 subunit‐specific agonist sensitizing activity of CNS4 on NMDA receptors expressed in the mammalian cells, we have carried out whole cell patch‐clamp electrophysiology assays using HEK293T cells transfected with GluN1/2A or 1/2AB receptor subunit constructs. Results from this experiment reveal that CNS4 insignificantly altered the peak current amplitude of 1/2A receptors (Ag, −323.5 ± 82 pA vs. +CNS4, −469.3 ± 152 pA, *n* = 4, *p* = .12), Figure 1A,B. However, CNS4 significantly reduced the peak current amplitude of 1/2AB receptors (Ag, −546 ± 32.34 pA vs. +CNS4–380.7 ± 36.61 pA, *n* = 8, *p* = .023). These results indicate that CNS4 reduces agonist efficacy in 1/2AB receptors when activated by saturating concentrations of agonists.

It was noticed from the traces that a transient and completely reversible peak was observed when CNS4 alone (in the external solution) was pre‐applied both in 1/2A and 1/2AB receptors (Figure 1A,C). This unexplained peak appeared when agonist was present in one of the lines of the perfusion system. Therefore, we have hypothesized that this peak resulted from leakage of agonist solution into the tip of the CNS4‐alone solution dispensing line or an artifact. However, this peak was reproducibly smaller with 1/2A receptors compared to 1/2AB receptors (Figure 1A,C). Indeed, this peak was as strong as the agonist‐induced peak in the 1/2AB receptors when the recording was repeated on the same cell multiple times. To re‐confirm that this peak appears only because of agonist spillover, we designed another experiment that did not contain an agonist‐alone dispensing line in the perfusion system. In this experiment, the perfusion system had only three active lines. (1) External (recording) solution, (2) 100 μM CNS4 alone, and (3) 100 μM CNS4 + 100 μM agonist solution. Other lines of the 8‐channel perfusion system were physically blocked with an endcap. We expected that, since there was no agonist‐alone line in this set of experiments, CNS4‐alone pre‐application should not generate an additional peak.

In contrast to the expectation, the peak still appeared in both 1/2A and 1/2AB receptor recordings (Figure 1E,G). The CNS4‐alone application‐induced transient current in 1/2A receptors was only 17.24 ± 1.6% of the CNS4+ agonist‐induced current (Figure 1I). However, in 1/2AB receptors, this peak was as high as 80.15 ± 2.3% of maximal current response. Nonetheless, these peaks were smaller than the ones that appeared when the agonist‐alone line was present (Figure 1C). And 100 μM CNS4 did not produce any measurable current response in untransfected HEK293T cells (Figure 1J). Therefore, currents induced by CNS4 alone are NMDA receptor‐mediated. This result indicates that CNS4 sensitizes ambient concentration of agonists in the 1/2AB subtype of NMDA receptors. Further, these observations corroborate increased glutamate potency in 1/2AB and 1/2A receptors in TEVC assay as previously reported.^40^

### 3.2 CNS4 alters glycine and glutamate efficacy in GluN2 subunit dependent manner

To further study the effect of CNS4 on agonist efficacy on all four GluN2 containing diheteromeric and 1/2AB triheteromeric receptors, TEVC electrophysiology assays were performed. Results obtained from these experiments reveal that CNS4 increases glycine efficacy in 1/2C and 1/2D and reduces glycine efficacy in 1/2B and 1/2AB subtypes of NMDA receptors (Figure 2A–E). The highest difference in efficacy was observed in 1/2D receptors, where 10 μM glycine produced 1.89 two‐fold higher (1.075 ± 0.07 vs. 2.04 ± 0.19) relative current response in the presence of 30 μM CNS4 compared to the experiments carried out in the absence of CNS4. CNS4 did not alter glycine efficacy in 1/2A receptors. In 1/2AB receptors, glycine efficacy reduction (2.37 ± 0.23 vs. 1.37 ± 0.08) was comparable to the 1/2B receptors (1.45 ± 0.13 vs. 0.88 ± 0.07).

In the converse experiment, glutamate dose–response curve was performed in the presence and absence of 30 μM CNS4, and 100 μM glycine was present in all solutions. Results from this set of experiments reveal that CNS4 reduced glutamate efficacy in 1/2AB and 1/2C receptors but did not alter glutamate efficacy in 1/2A, 1/2B and 1/2D receptors (Figure 3A–E). The biggest difference was observed in 1/2AB receptors, where CNS4 reduced glutamate efficacy to 5.3 fold (14.85 ± 2.06 vs. 2.79 ± 0.43), although no significant change was observed in 1/2A (3.45 ± 0.27 vs. 2.95 ± 0.45) or 1/2B (4.91 ± 0.52 vs. 4.27 ± 0.82) diheteromeric receptors. Overall, CNS4 significantly reduced glutamate efficacy in 1/2AB and 1/2C receptors.

### 3.3 CNS4 does not alter competitive antagonists but alters uncompetitive NMDA receptor antagonist activity

**FIGURE 4:**
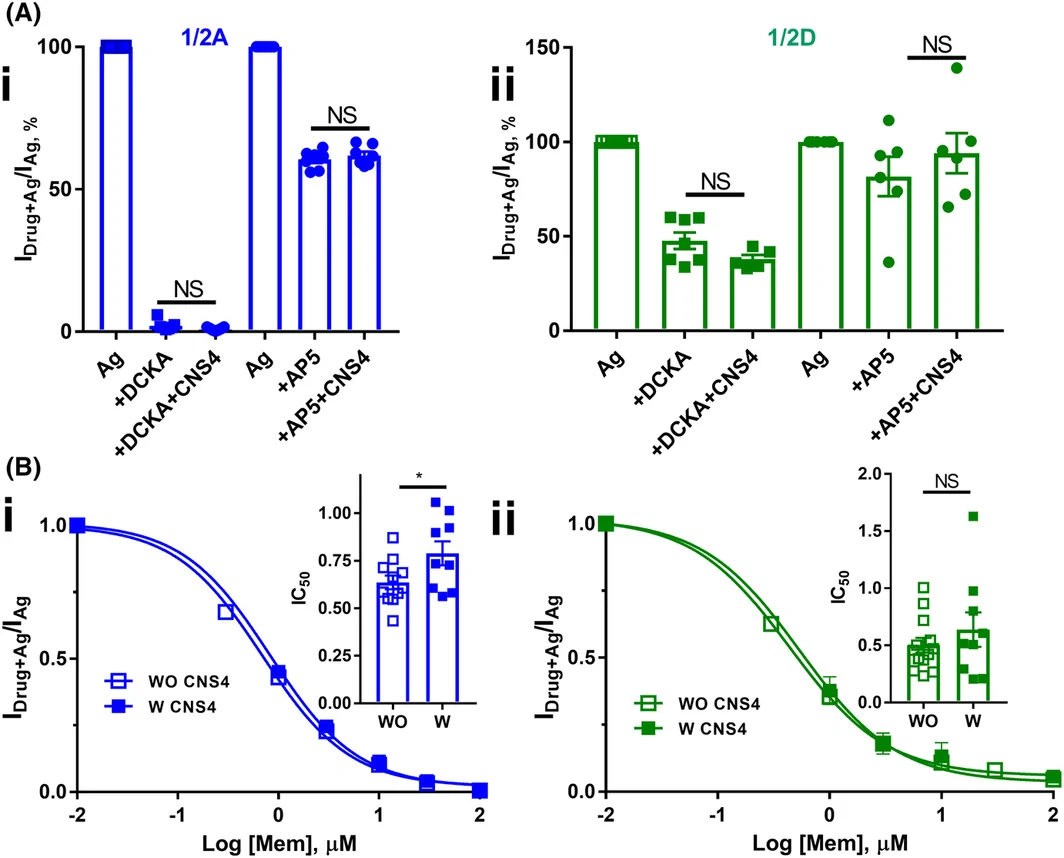
Effect of CNS4 on competitive and uncompetitive NMDA receptor antagonists. (A) 1/2A or 1/2D receptors are activated by 100 μM agonists (glutamate and glycine) and followed by 100 μM DCKA and then DCKA plus 100 μM CNS4. A similar experiment was performed with 100 μM DL‐AP5. CNS4 did not affect DCKA efficacy in blocking 1/2A currents (DCKA, 97.85 ± 0.66, *n* = 7 (7) and DCKA plus CNS4 98.98 ± 0.21% *n* = 7 (7), *p* = .1016). Similarly DL‐AP5 blocked 39.53 ± 1.22 and DL‐AP5 plus CNS4 blocked 38.10 ± 1.28%, *n* = 7 (7), *p* = .182 of 1/2A currents. 1/2D receptors CNS4 did not affect DCKA (DCKA, 52.30 ± 0.4.41 and DCKA plus CNS4 62.09 ± 2.27%, *n* = 5 (5), *p* = .3133) or DL‐AP5 (DL‐AP5, 18.28 ± 10.47 and, DL‐AP5 plus CNS4 5.96.10 ± 10.59%, *n* = 6 (5), *p* = .4750) efficacy in blocking 1/2D currents. Paired student‐*t* test was performed to calculate the statistical significance. (B) Memantine dose response curves performed on 1/2A and 1/2D receptors in the without (WO) and with (W) of 30 μM CNS4. Histograms represent the IC_50_ values for 1/2A receptors (WO, 0.64 ± 0.03, *n* = 11 (10) vs. W, 0.87 ± 0.09 μM, *n* = 10 (8) *p* = .0407) and 1/2D receptors (WO, 0.50 ± 0.06, *n* = 13(10) vs. W, 0.64 ± 0.15 μM, *n* = 9 (7) *p* = .3645). I_Drug_ = CNS4 or CNS4 + DCKA or CNS4 + DL‐AP5 or CNS4 + Memantine. Unpaired student‐*t* test was performed to calculate the statistical significance. *p < .05; NS, not significant.

The reduction in glycine and glutamate efficacy directed us to study the influence of CNS4 on glycine and glutamate site competitive antagonists, DCKA and DL‐AP5 respectively. If CNS4 directly competes with GluN1 or GluN2 competitive antagonists or indirectly influences the activity, there should be a change in DCKA and/ or DL‐AP5 activity in blocking agonist‐induced currents. For this set of experiments, we have chosen 1/2A and 1/2D subtypes, since these subtypes have differences in various physiological properties including agonist potency, deactivation time course, and ion permeability.^20^ In order to test our hypothesis, 1/2A and 1/2D receptors were activated by 100 μM agonists (glutamate and glycine) and we then applied a single dose of 100 μM DCKA or 100 μM DL‐AP5 in the absence and presence of 100 μM CNS4. Agonist‐induced maximal activation was normalized to 100%, and relative inhibition of DCKA and DL‐AP5 was calculated. Remarkably, neither DCKA nor DL‐AP5 activity was altered by 100 μM CNS4 (Figure 4) in 1/2A and 1/2D receptors. The glycine site antagonist DCKA blocked 97.85 ± 0.66% and 98.98 ± 0.21% of 1/2A currents in the presence and absence of CNS4, respectively. On the other hand, glutamate site antagonist DL‐AP5 blocked 1/2A currents 39.53 ± 1.22 and 38.10 ± 1.28% in the absence and presence of CNS4, respectively. On 1/2D receptors, DCKA blocked 52.30 ± 0.4.41 and 62.09 ± 2.27% of agonist‐induced currents in the presence and absence of CNS4, respectively. DL‐AP5 blocked 5.96 ± 10.59 and 18.28 ± 10.47% in the presence and absence of CNS4, respectively. These observations suggest that CNS4 does not interfere with glycine or glutamate site antagonist activity in both 1/2A and 1/2D receptors.

Next, we studied the effect of CNS4 on activity of the uncompetitive NMDA receptor blocker, memantine. A memantine dose–response curve was performed in the presence and absence of 30 μM CNS4. Results from this assay reveal that CNS4 made a minimal but statistically significant reduction in memantine potency (IC_50_ 0.64 ± 0.03 vs. 0.87 ± 0.09 μM) in the 1/2A receptor, while memantine potency on 1/2D receptors remained unaltered (IC_50_ 0.50 ± 0.06 vs. 0.64 ± 0.15 μM), Figure 4B.

### 3.4 CNS4 preferentially modulates agonist‐induced current directions in 1/2A receptors compared to 1/2D receptors

The changes in memantine potency in 1/2A receptors directed us to study the effect of CNS4 on voltage dependence through a current–voltage (I‐V) relationship assay. To identify the extracellular calcium concentration‐induced changes in reversal potential (E_rev_), two different concentrations of Ca^2+^ were studied (1.8 mM [normal] and 0.018 mM [low]). Further, to demonstrate the effect of CNS4 on Na^+^ ion movement through the NMDA receptor channel, NaCl was replaced with a membrane impermeable compound, NMDG (referred to as Na_free_). And 100 μM glycine and 100 μM glutamate were used as agonists to activate the receptor.

**FIGURE 5:**
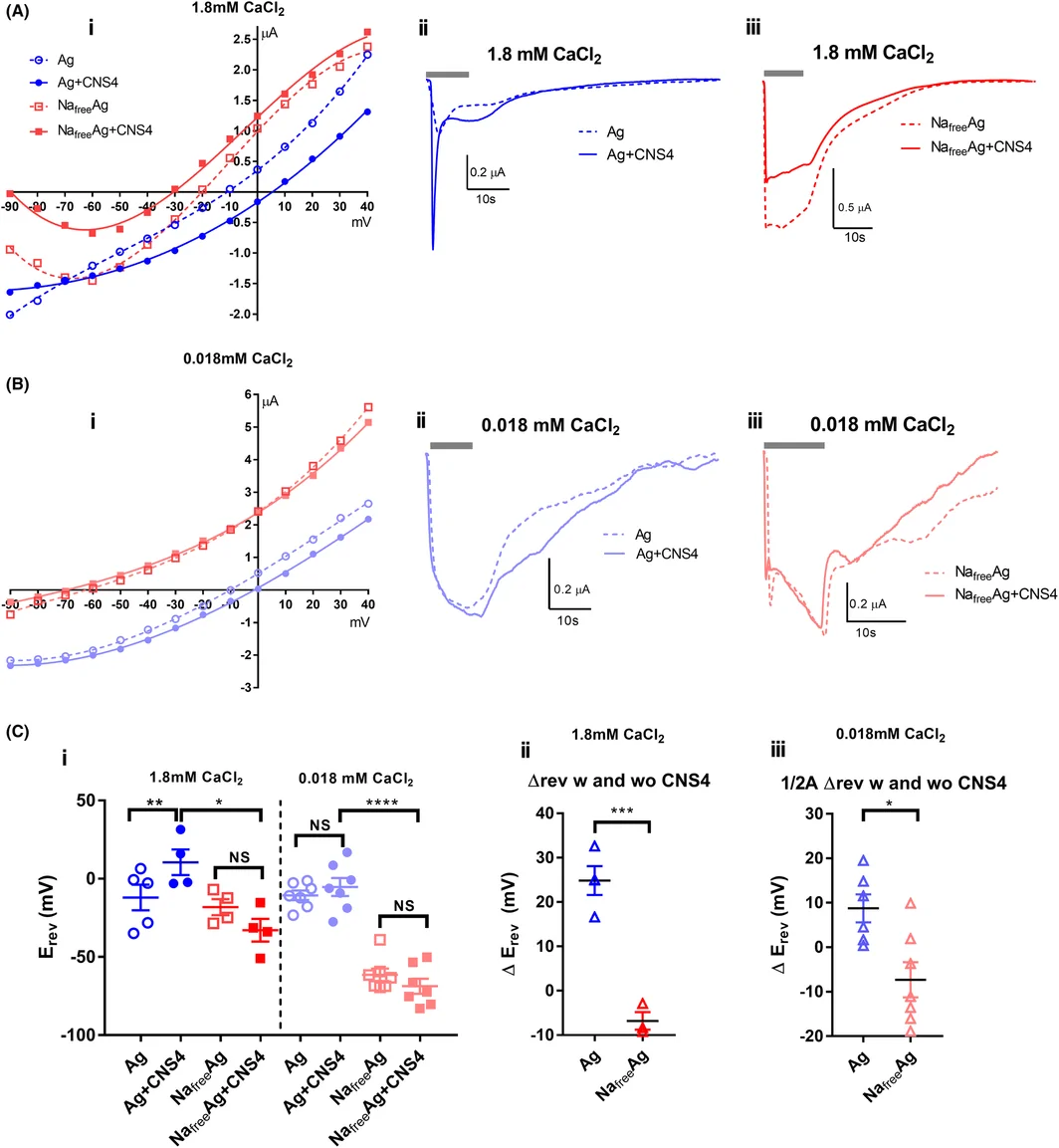
Effect on CNS4 on agonist induced current directions in 1/2A receptors. Current –Voltage (I‐V) relationship of 1/2A receptors was studied with normal (A) and low (B) CaCl2 containing extracellular solutions. 100 μM glutamate and 100 μM glycine was used as agonist to activate the receptors at −90 mv and performed voltage ramp until +40 mV at 10 mV intervals. (C) Comparison of reversal potentials values. Dark and light blue circles indicate normal and low CaCl_2_ experiments, respectively. Dark and light red squares indicate equivalent I‐V experiments with NMDG (Na_free_). Dotted and thick lines denote the absence (empty circles or squares) and presence (filled circles or squares) of 100 μM CNS4, respectively. Traces (Ai&ii, Bi&ii) represent the current response at −90 mv. *N* = 5 (5) & *n* = 7(7) for normal & low Ca^2+^ assays, respectively. E_rev_ obtained from each oocyte with different agonists were compared in a pairwise manner. Paired student *t*‐test was performed to compare the data presented at Ci. Unpaired student's *t*‐test was performed to compare the difference in reversal potentials (triangles). *p < .05, **p < .01, ***p < .001, ****p < .001.

Results from 1.8 mM (normal) Ca^2+^ experiments on 1/2A receptors reveal that CNS4 significantly alters the E_rev_ (Figure 5A,Ci). CNS4 converted the negative E_rev_ of agonist‐induced 1/2A receptor currents to a positive E_rev_. The E_rev_ without (WO) and with (W) 100 μM CNS4 were: −12.05 ± 8.18, *n* = 5, vs 10.49 ± 8.23 mV, *n* = 4, *p* = .004. Data acquired from the experiments conducted with Na_free_ solution indicate no significant changes in 1/2A current E_rev_. The E_rev_ were: WO, −18.16 ± 5.09, *n* = 4, versus W, −32.93 ± 7.03 mV, *n* = 4, *p* = .16. Next, we calculated the differences in the reversal potentials (∆E_rev_) between WO and W CNS4 of Na^+^ and Na_free_ data sets and compared them. A pairwise comparison was performed for E_rev_ obtained from different solutions on each oocyte studied. These differences (∆E_rev_ with Na^+^, 24.85 ± 3.26, *n* = 4 vs. Na_free_, −6.79 ± 1.98 mV, *n* = 3, *p* = .007) were highly significant (Figure 5Cii). Further, more negative membrane potentials (−70 mV and less) exhibited a strong inhibition of inward current, and less negative potentials (−50 mV and more) started outward rectifying. With the Na_free_ solutions, both in the presence and absence of CNS4, maximum inward current was observed at −60 mV, as it was with the Na^+^ solution (Figure 5A). Voltage dependent changes with inward current observed in the Na_free_ solutions resembled the effects reported in the presence of Mg^2+^ in the 1/2A receptors.^40^

The equivalent set of experiments performed with low Ca^2+^ concentration revealed no changes in the E_rev_ when compared between WO and W CNS4 data sets (Figure 5B,C). The E_rev_ of experiments with Na^+^ were: WO ‐10.63 ± 3.03, *n* = 7 versus W, −32.93 ± 7.3 mV, *n* = 7, *p* = .42. For Na_free_ solutions: WO −61.38 ± 3.99, *n* = 7 versus W, −68.71 ± 4.82 mV, *n* = 7, *p* = .113. However, the ∆E_rev_ between W and WO CNS4 of Na^+^ and Na_free_ experiments were significant (∆E_rev_ with Na^+^, 8.76 ± 3.15, *n* = 6, vs Na_free_, −7.32 ± 3.95 mV, *n* = 7, *p* = .01) (Figure 5Ciii). Finally, comparing the E_rev_ of experiments done with and without Na^+^ revealed that CNS4 significantly increased inward currents (positive or less negative E_rev_) of 1/2A receptors in the presence of Na^+^, at both normal (10.49 ± 8.24, *n* = 4 vs. −32.94 ± 7.30 mV, *n* = 4, *p* = .043) and low (−5.32 ± 5.76, *n* = 7, vs. −68.71 ± 4.82 mV, *n* = 7, *p* = .0001) Ca^2+^ levels (Figure 5Ci). Notably, in both normal and low Ca^2+^ experiments carried out with Na^+^, E_rev_ were comparable in the absence of CNS4 (−12.05 ± 8.18 and − 10.63 ± 3.03 mV) (Figure 5Ci).

**FIGURE 6:**
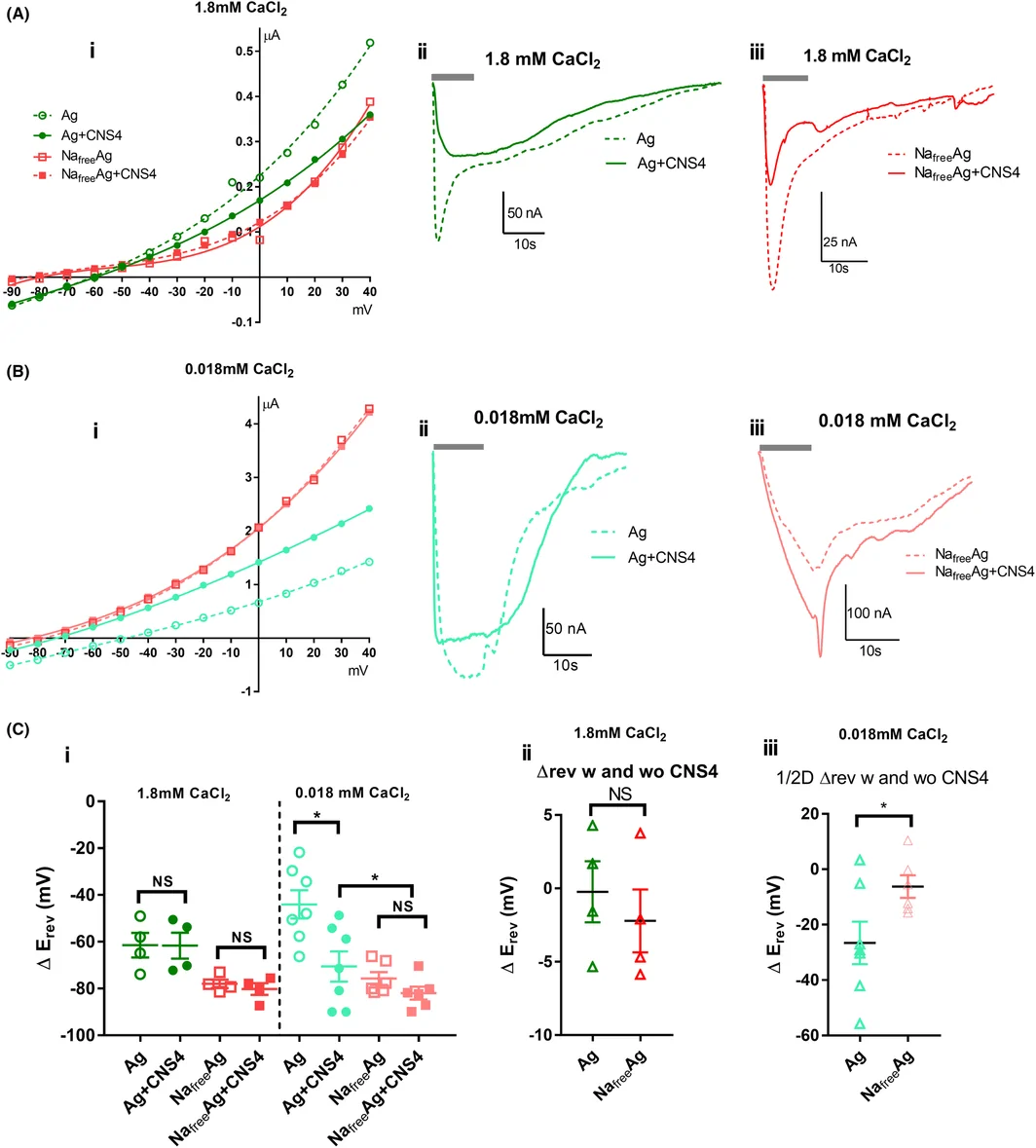
Effect on CNS4 on agonist induced current directions in 1/2D receptors. Current –Voltage (I‐V) relationship of 1/2D receptors was studied with normal (A) and low (B) CaCl2 containing extracellular solutions. 100 μM glutamate and 100 μM glycine was used as agonist to activate the receptors at −90 mv and performed voltage ramp until +40 mV at 10 mV intervals. Dark and light green circles indicate normal and low CaCl_2_ experiments, respectively. (C) Comparison of reversal potentials values. Dark and light red squares indicate equivalent I‐V experiments with NMDG (Na_free_). Dotted and thick lines denote the absence (empty circles or squares) and presence (filled circles or squares) of 100 μM CNS4, respectively. Traces (Ai&ii, Bi&ii) represent the current response at −90 mv. *N* = 4 (4) & *n* = 7(7) for normal & low Ca^2+^ assays respectively. E_rev_ obtained from each oocyte with different agonists were compared in a pairwise manner. Paired student *t*‐test was performed to compare the data presented at Ci. Unpaired student's *t*‐test was performed to compare the difference in reversal potentials (triangles). *p < .05.

Similar I‐V relationship experiments were carried out on 1/2D receptors. Results from normal Ca^2+^ experiments on 1/2D subunit revealed that CNS4 did not alter the E_rev_ (Figure 6A,C). E_rev_ with normal calcium were: WO, −61.43 ± 5.26, *n* = 4, versus W, −61.66 ± 5.55 mV, *n* = 4, *p* = .919. Also, data from the experiments conducted with the Na_free_ solution indicate no significant changes in 1/2D current direction. E_rev_ were: WO, −77.99 ± 1.77, *n* = 4, versus W, −80.20 ± 2.54 mV, *n* = 4, *p* = .378. Next, we calculated the ∆E_rev_ between WO and W CNS4 of Na^+^ and Na_free_ data sets and compared them. ∆E_rev_ with Na^+^, −0.22 ± 2.08, *n* = 4 vs Na_free_, −2.21 ± 2.14 mV, *n* = 4, *p* = .531 were not significant (Figure 6Cii).

Additional experiments with low Ca^2+^ revealed a significant reduction in E_rev_ when compared with WO and W CNS4 data sets (Figure 6B,Ci). E_rev_ of experiments with Na^+^ were, WO ‐44.02 ± 6.04, *n* = 7, vs W, −70.57 ± 6.44 mV, *n* = 7, *p* = .013. Experiments with Na_free_, however, showed no difference between WO and W CNS4 experiments (WO ‐61.38 ± 3.99, *n* = 7 vs. W, −68.71 ± 4.82 mV, *n* = 7, *p* = .113). The ∆E_rev_ between W and WO CNS4 of Na^+^ and Na_free_ experiments were significant (∆E_rev_ potential with Na^+^, −26.55 ± 7.66, *n* = 7, vs. Na_free_, −6.22 ± 4.08 mV, *n* = 6, *p* = .048) (Figure 6Ciii). Finally, comparing the E_rev_ of experiments done with and without Na^+^ revealed that CNS4 did not significantly alter the E_rev_ of 1/2D receptors at normal Ca^2+^ (−61.66 ± 5.55, *n* = 4 vs. −80.21 ± 2.54 mV, *n* = 4, *p* = .063). However, in low Ca^2+^ experiments, E_rev_ were significantly reduced (−70.57 ± 6.44, *n* = 7, vs. −81.93 ± 2.74 mV, *n* = 6, *p* = .043) in the absence of Na^+^ (Figure 6Ci). This reveals that at low Ca^2+^, CNS4 blocks the permeable Na^+^ ions. However, interestingly, in experiments with low Ca^2+^, 1/2D receptors produced visibly larger steady state currents compared to the normal Ca^2+^, as seen in the traces (Figure 6Bii & iii).

Collectively, the results of experiments I‐V indicate that CNS4 preferentially alters E_rev_ of 1/2A currents to less negative (or positive) in the presence of Na^+^, at normal Ca^2+^ levels. In the absence of Na^+^, E_rev_ remained unchanged in both 1/2A and 1/2D receptors, indicating that CNS4 is primarily modulating Na^+^ inward currents. Comparison of ∆E_rev_ revealed that Na_free_ experiments were less impacted by CNS4 at both normal and low Ca^2+^ levels in both 1/2A & 1/2D receptors (Figures 5Cii&iii and 6Cii&iii). However, in 1/2D receptors, ∆E_rev_ were less negative at low Ca^2+^ levels with Na_free_ solutions, unlike 1/2A receptors, where equivalent ∆E_rev_ were more negative (Figure 5Cii&iii), indicating that CNS4 maintained inward currents until the membrane potential was closer to zero (−6.22 ± 4.08 mV) in 1/2D receptors at low Ca^2+^ and Na_free_ conditions. A transient peak that occurs soon after stopping agonist application shows disinhibition of CNS4 effect (Figure 6Biii).

### 3.5 Role of GluN1/2A ABD interface in determining the modulatory effect of CNS4

NMDA receptor allosteric modulators bind at various locations in the extracellular NTD or ABD interface or at pre‐helices formed around the channel pore, as recently reviewed.^41^ CNS4 did not affect the activity of ifenprodil on 1/2A, 1/2B & 1/2AB receptors.^40^ This directed us to explore the effect of CNS4 on the GluN1/2A ABD interface mutations. We have studied the effect of five GluN1 mutants by co‐expressing with wild type GluN2A subunit. These mutations are located at site‐I (N521A and N521D), site‐II (K531A, Y535A) and site‐III (E781A) of the intersubunit interaction sites of GluN1 subunit ABD.^42^

**FIGURE 7:**
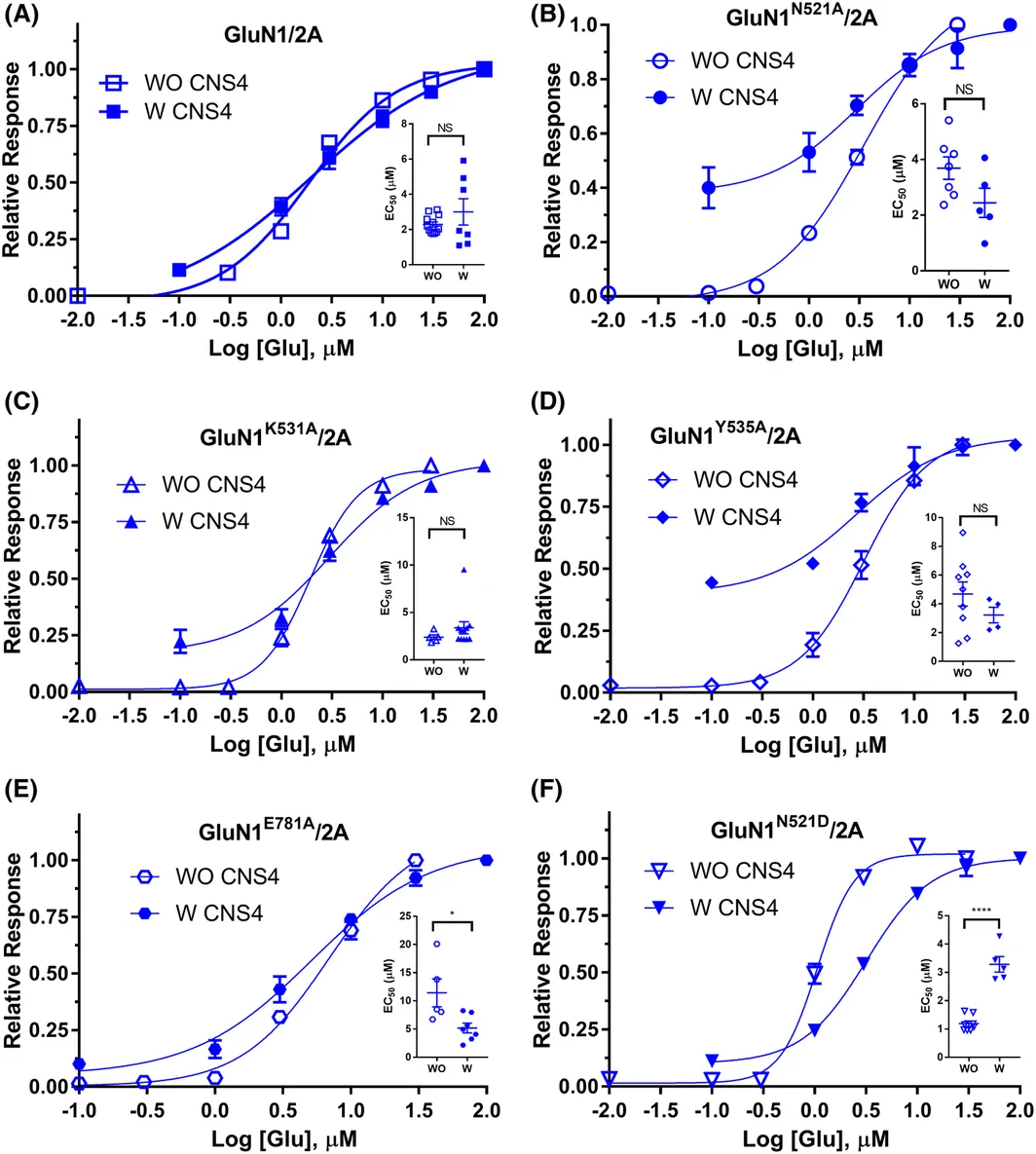
GluN1/2A ABD interface interaction disrupting mutations positively and negatively modulate CNS4 potency. Glutamate dose–response curves were performed without (empty shapes) and with (filled shapes) of 30 μM CNS4 on the following GluN1 mutants (N521A, N521D, K531A, Y535A and E781A) co‐expressed with wild type GluN2A subunit. 100 μM glycine was kept constant. Dot plots summarize the EC_50_ values. Glutamate potency changes WO versus W CNS4 is compared: 1/2A (2.29 ± 0.14, *n* = 12 (5) vs. 3.00 ± 0.74, *n* = 7(6), *p* = .24), N521A_1/2A (3.69 ± 0.40, *n* = 7(3) vs. 2.44 ± 0.52, *n* = 5(3), *p* = .084), K531A_1/2A (2.4 ± 0.24, *n* = 5 (4) vs. 3.38 ± 0.63, *n* = 11(5), *p* = .33), Y535A_1/2A (4.69 ± 0.83, *n* = 9 (4) vs. 3.22 ± 0.54, *n* = 4(3), *p* = .29), E781A_1/2A (11.43 ± 2.48, *n* = 5(3) vs. 5.18 ± 0.86, *n* = 7(4), *p* = .021, N521D_1/2A (1.19 ± 0.09, *n* = 8 (3) vs. 3.2 ± 0.27, *n* = 5(3), *p* < .0001). Values are average ± SEM. Unpaired student's *t*‐test, *p* < .05. *p < .05, ****p < .0001. Note: WO CNS4 glutamate dose response data was obtained from the previously published work.^43^

**FIGURE 8:**
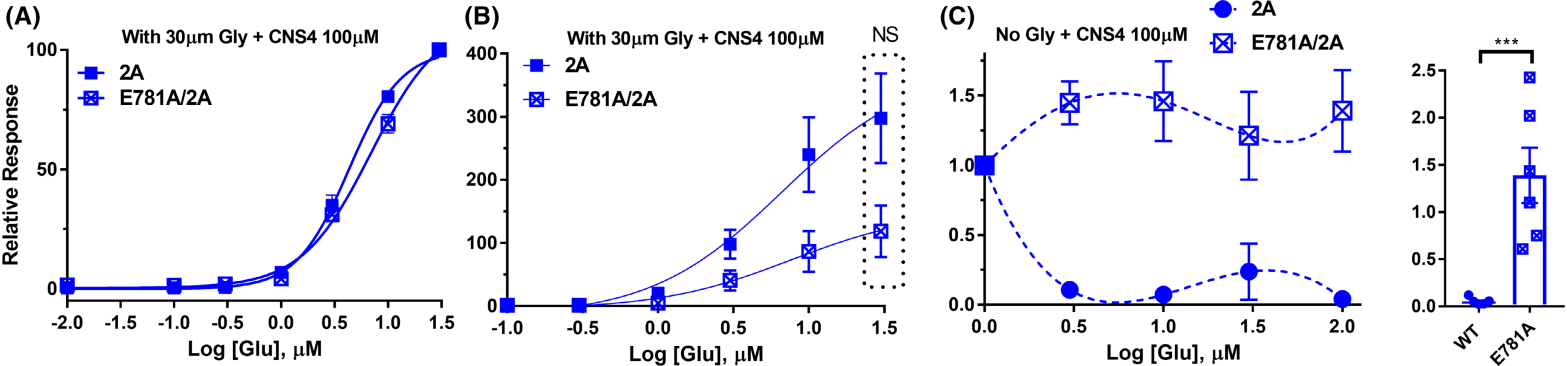
CNS4 increases glutamate efficacy in the absence of co‐agonist glycine on E781A_1/2A mutant receptor. Glutamate dose–response curves were performed in the wild type and E781A mutant GluN1 co‐expressed with wild type GluN2A subunit. No changes in glutamate potency (A) (4.42 ± 0.36, *n* = 9(3) vs. 5.45 ± 0.53 μM, *n* = 5(4), *p* = .74), or efficacy (B) (297.8 ± 71.06, *n* = 9 (3) vs. 118.5 ± 40.87 fold, *n* = 5 (4), *p* = .11), observed in the presence of 30 μM glycine and 30 μM CNS4. However, in the absence of glycine but presence of CNS4, glutamate efficacy (C) was ~34 fold different between wild type 1/2A receptor and E781A_1/2A receptor at 100 μM glutamate induced relative current amplitude (1/2A, 0.04 ± 0.02, *n* = 6(3) vs. E781A_1/2A 1.39 ± 0.29 fold, *n* = 6 (3), *p* = .001). Values are average ± SEM. Unpaired student's *t*‐test, ***p < .001.

Results from the glutamate dose–response curves with and without 30 μM glutamate reveal that CNS4 did not significantly alter glutamate potency on N521A, K531A, and Y535A mutant receptors (Figure 7A–F). Nonetheless, at lower glutamate concentrations CNS4 numerically improved glutamate potency in all three (Site‐I&II) mutations (Figure 7B–D). E781A_1/2A is the only mutant receptor where CNS4 increased glutamate potency (Figure 7E). Further, we compared the glutamate potency and efficacy of wild type 1/2A and E781A_1/2A mutant receptors in the presence of CNS4. Neither of these was significantly different (Figure 8). However, interestingly, in the absence of co‐agonist glycine and presence of CNS4, 100 μM glutamate efficacy was ~34‐fold higher in the E781A mutant compared to wild type 1/2A receptors (1/2A, 0.04 ± 0.02 vs. E781A 1.39 ± 0.29 fold, *p* = .001) (Figure 8). The E781A mutation is at Site‐III of GluN1/2A ABD interface, that spans around the distal end of the ABD heterodimer, preceding the pre‐M1 helix positioned parallel to the lipid bilayer.^42^, ^44^

Remarkably, neither glycine nor glutamate potency was altered in E781A_1/2A receptors.^43^ In contrast, another mutation at the N521 position, N521D, increased both glycine and glutamate potency compared to the wild type 1/2A receptor, as previously reported.^43^ Therefore, we studied the effect of CNS4 on the N521D mutation. Interestingly, CNS4 significantly reduced glutamate potency (WO, 1.19 ± 0.09, vs. W, 3.2 ± 0.27 μM, *p* < .0001) on N521D_1/2A receptors (Figure 7F). Negative (N521D) and positive (E781A) modulation of glutamate potency at Site‐I and III mutants (respectively) indicate that CNS4 activity stabilizes or disrupts the ABD interface interactions based on the amino acid sidechains at these sites.

## 4 DISCUSSION

In the present study, we have carried out a pharmacological characterization of CNS4 which is known to potentiate 1/2C and 1/2D subtype of NMDA receptors at low glutamate (0.3 μM) concentration.^40^ In the patch clamp experiments, the reproducible occurrence of a reversible peak produced by CNS4 with no agonist indicates ambient agonist sensitization (Figure 1). The differences in the current amplitude in 1/2A & 1/2AB (17.24 ± 1.6 vs. 80.15 ± 2.3%) reveal the subunit preference in this modulatory effect. Remarkably, three out of four subunits in 1/2AB and 1/2A receptors are essentially identical. Thus, 1/2AB is the closest family member of the 1/2A receptor. Despite this high homology, CNS4 differentiates the function of these subunits in an agonist concentration‐dependent manner.

A notable reduction of both glycine and glutamate efficacy at 1/2AB receptors indicates that in the presence of CNS4, agonists maximally activate the 1/2AB receptors at lower than 1 μM, as previously reported.^40^ Thus, higher agonist concentrations could not further increase the already maximized current response (Figures 2 and 3). This was not seen with either agonist at 1/2A receptor, and seen only with glycine in 1/2B receptors, indicating that CNS4 could dissect the diheteromeric from triheteromeric receptors which are predominantly expressed throughout the hippocampus and cortex.^12^, ^13^, ^14^, ^15^, ^16^, ^17^ A substantial increase in glycine efficacy in both 1/2C & 1/2D (1.8 & 1.9 fold, Figure 2), a reduction in glutamate efficacy at 1/2C (2.1 fold), and an insignificant effect in 1/2D (0.8 fold) again indicates subunit selective activity of CNS4 on two highly homologous GluN2 subtypes of NMDA receptor. Had CNS4 reduced glutamate affinity at higher glutamate concentrations, one or both of the competitive antagonists studied (DCKA & DL‐AP5) should have blocked NMDA receptors better in the presence of CNS4. But this was not observed in 1/2A or 1/2D receptors (Figure 4). We chose a less potent racemic mixture of AP5, DL‐AP5,^45^ with an expectation that even if CNS4 produced a mild effect, there would be enough room to notice this effect. Because DL‐AP5 blocks only ~39% and ~18% of current in 1/2A and 1/2D receptors, there was enough current left to block (or potentiate by unblocking), if CNS4 had such a power to exercise. In contrast, CNS4 had no effect on DL‐AP5 activity (Figure 4A). This reaffirms that CNS4 is an allosteric modulator, and has no effect even on a weak orthosteric antagonist activity.

As an allosteric modulator, CNS4 was not expected to affect the activity of uncompetitive antagonist memantine that binds at the extracellular vestibule of the NMDA receptor channel. On the contrary, it minimally but significantly reduced (0.64 vs. 0.87 μM IC_50_) memantine potency at 1/2A receptors. This motivated us to carry out the I‐V studies to determine the effect of CNS4 on the E_rev_ of permeant cations. The data from I‐V experiments show that in the 1/2A receptors, CNS4 produced two extreme E_rev_ in the presence and absence of Na^+^ (10.49 ± 8.23 and −32.93 ± 7.03 mv) (Figure 5A–C). Both normal and low Ca^2+^ experiments demonstrated this phenomenon. Notably, 1/2A subtype of NMDA receptors are permeable to both Ca^2+^ and Na^+^.^41^, ^46^ Had CNS4 not potentiated Na^+^ currents, there should not be a strong reduction in inward current (with more negative E_rev_) that was observed in the absence of Na^+^. If CNS4 potentiated the Ca^2+^ currents in 1/2A receptors, at least in the experiments with normal Ca^2+^, again there should not have been a reduction in E_rev_ while CNS4 was present. However, we observed a less negative E_rev_ (WO, −18.16 vs. W, −32.93 mv). These findings indicate that CNS4, when activated by 100 μM agonists, preferentially potentiates Na^+^ currents in 1/2A receptors. CNS4‐induced changes in E_rev_ preferentially occurring at 1/2A receptors explain why memantine potency was altered in 1/2A but not in 1/2D receptors. Remarkably, despite the different permeant cations used in these two assays (300 μM BaCl_2_ for memantine dose–response assay [Figure 4] & 1.8 mM Ca^2+^ for I‐V assay, [Figure 5]), the results consistently indicate the influence of CNS4 on the 1/2A receptor channel.

GluN1 K531A, Y535A and E781A mutants made memantine more potent, while N521A & N521D had no effect.^43^ However, memantine is an uncompetitive NMDA receptor antagonist binding at the extracellular vestibule of the channel pore.^47^ So, drugs binding at locations downstream from the ABD interface also can alter the 1/2A ABD interface interactions. Therefore, positive and negative modulatory effects seen with E781A and N521D are not necessarily confirming that CNS4 is binding at the ABD interface. Further mutagenesis or cryo‐EM studies are needed to identify the CNS4 binding site. The visual abstract illustrates the mechanism of action of CNS4 that we hypothesize based on the observations and inference made from these results. This needs to be experimentally evaluated.

Regardless of the binding site, as long as a compound could improve agonist potency, it could be useful for the treatment of GRIN disorders evolving from loss of function GRIN mutations. Currently, we are generating clinically reported GRIN mutations to study the effect of CNS4 and related compounds for their ability to improve the function of mutant receptors. Further, CNS4 could be useful to pharmacologically isolate native 1/2AB triheteromeric receptor currents from 1/2A and 1/2B diheteromeric receptors in neurons. Based on the present study, ~1 μM glutamate almost maximally activates the 1/2AB triheteromeric receptor in the presence of 30 μM CNS4. Any increase in current response with higher concentrations of glutamate should be coming from 1/2A and 1/2B diheteromeric receptors. This might also include any 1/2C and 1/2D currents. But these currents can be blocked with a 2C & 2D subunit selective competitive antagonist, UBP791.^48^ Thus, CNS4 can help estimate an approximate percentage of 1/2AB triheteromeric receptor currents from an animal brain tissue. In conclusion, CNS4 and its future analogs will serve as potential lead compounds to develop drugs for the treatment of neuropsychiatric conditions evolving from hypoglutamatergic conditions. Also, could be useful as chemical tools to study native NMDA receptors.

Sensitization of ambient levels of agonists in 1/2AB triheteromeric receptors, specific changes in glycine and glutamate efficacy, and modulation of permeant cation at GluN1/2 subtype are novel findings resulting from the pharmacological characterization conducted in this study. Additionally, we have identified the role of the E781 amino acid position in the activity of CNS4. This finding adds an important piece to the puzzle of understanding the molecular basis of CNS4's mechanism of action and provides insight for future drug development efforts. However, it is important to acknowledge the limitations of our study. We were unable to perform higher resolution electrophysiology assays and co‐crystallization of CNS4 with one of the GluN1/2 receptors due to constraints such as lack of expertise and resources. While these experiments would have provided deeper insights into the molecular mechanisms involved, their absence does not undermine the significance of our findings. Instead, it highlights avenues for future collaborative work to address these gaps in knowledge.

To bridge the gap between a hit compound and a lead compound, we are currently engaged in pharmacokinetics work. This ongoing research aims to determine crucial pharmacokinetic parameters, including drug absorption, bioavailability, time taken to reach maximum plasma concentration, and blood–brain barrier penetration. The knowledge gained from these studies will be instrumental in optimizing CNS4 and its congeners to transform them into clinically valuable drug candidates. Overall, the results presented in this manuscript contribute novel insights into CNS4 pharmacology and its allosteric modulation. While some limitations exist, we believe that our findings pave the way for further investigations and potential therapeutic applications of CNS4.

## AUTHOR CONTRIBUTIONS

Blaise M. Costa contributed to conceptualization, project administration, and manuscript writing. Blaise M. Costa and Pamela J. VandeVord contributed to funding acquisition and progress reports. Blaise M. Costa, Seth C. Boehringer, Lina Cortez Kwapisz and Tulia V. Johnston contributed to the experiments. Blaise M. Costa, Seth C. Boehringer, Lina Cortez Kwapisz & Tulia V. Johnston contributed to data analysis.

## FUNDING INFORMATION

This work was originally funded by American Heart Association Scientist Development Grant (16SDG27480023) funded to BMC; and partially funded by the VCOM and Virginia Tech Institute for Critical Technology and Applied Science REAP Grant funded to BMC and PJV.

## SIGNIFICANCE

CNS4 modulates NMDA receptor function based on glutamate concentration and GluN2 subunit composition. This modulatory effect arises from changes in agonist efficacy and extracellular Ca^2+^ concentration‐dependent changes in cation influx through the NMDA receptor channel. Overall, CNS4 exhibits a glutamate receptor optimizer‐like activity.

## Data Availability

The authors declare that all the data supporting the findings of this study are contained within the paper.
